# Transient Cortical Blindness After Coronary Angiography, Bypass Graft Angiography, and Coronary Angioplasty

**DOI:** 10.7759/cureus.12542

**Published:** 2021-01-07

**Authors:** Zhen-Vin Lee, Ramesh Singh Arjan Singh

**Affiliations:** 1 Cardiology Unit, Department of Medicine, University Malaya Medical Centre, Kuala Lumpur, MYS

**Keywords:** transient cortical blindness, coronary angiography, bypass graft angiography, coronary angioplasty, bilateral blurring of vision, contrast agent, complication

## Abstract

Transient cortical blindness after coronary angiography has long been reported in the literature; however, this condition remains rare until today. We report a case of transient cortical blindness after coronary angiography, bypass graft angiography, and coronary angioplasty, which was deemed to be secondary to contrast agent. A 60-year-old man who underwent prior coronary artery bypass grafting (CABG) started to experience recurrence of exertional and resting chest pain one year after CABG. In addition to coronary artery disease, he has underlying type 2 diabetes mellitus, hypertension, and dyslipidemia. Due to technical reasons, he was unable to undergo a computed tomography (CT) angiography of the coronary arteries and bypass grafts. Invasive coronary and bypass graft angiography were done, followed by stenting of the left circumflex artery. Thirty minutes after completion of the procedure, the patient had bilateral blurring of vision, which worsened drastically to only being able to perceive light bilaterally. The patient otherwise did not have any other neurological deficits. Binocular indirect ophthalmoscopy revealed no significant abnormalities apart from mild non-proliferative diabetic retinopathy of the left eye. A non-contrasted CT scan of the brain revealed acute subarachnoid bleed in both occipital lobes, but a subsequent magnetic resonance imaging scan of the brain revealed no evidence of intracranial bleed. The patient’s vision gradually improved eight hours after the index event, and his vision completely normalized 12 hours later. The patient was discharged well two days later, and at one-month, three-month, and six-month follow-up, the patient remained angina-free, and his vision had remained stable bilaterally.

## Introduction

Cortical blindness refers to the loss of vision produced by lesions affecting geniculocalcarine visual pathways [1]. Transient cortical blindness secondary to angiography (cerebral, vertebral, and coronary) has long been reported in the literature, and many different mechanisms have been postulated. The exact pathophysiology of transient cortical blindness, however, remains unknown until today. We report a case of transient cortical blindness, a surprising complication for doctors and distressful to patients. This event happened after coronary angiography, bypass graft angiography, and coronary angioplasty.

## Case presentation

A 60-year-old man with underlying coronary artery disease, type 2 diabetes mellitus, hypertension, and dyslipidemia presented complaining of frequent exertional and resting chest pain. He underwent a coronary artery bypass grafting (CABG) one year prior in which four bypass grafts were implanted, namely, left internal mammary artery (LIMA) to left anterior descending (LAD) artery, saphenous vein graft (SVG) to diagonal artery, SVG to obtuse marginal artery, and SVG to posterior descending artery. Due to technical reasons, he was unable to undergo a computed tomography (CT) angiography of the coronary arteries and bypass grafts and was subjected to an invasive coronary and bypass graft angiography, which was performed via the right femoral artery access. Angiography of the native coronary arteries revealed 80% stenosis of mid-LAD, chronic total occlusion (CTO) of proximal left circumflex artery, 90% stenosis of proximal right coronary artery (RCA), and 70% stenosis of mid-RCA. Bypass graft angiography revealed patent SVG to diagonal artery and patent SVG to posterior descending artery. There was, however, severe difficulty in engaging the LIMA to LAD bypass graft, which resulted in an increase in contrast amount and procedural time. The LIMA to LAD bypass graft was subsequently successfully engaged and was noted to be patent. The SVG to obtuse marginal artery bypass graft was unable to be located and was deemed to be completely occluded. Hence, a decision was then made to perform angioplasty to the left circumflex artery, which was uneventful. One drug-eluting stent was implanted in the proximal left circumflex artery. A total of 400 mL of non-ionic, low-osmolar iodinated contrast (iopamidol) and 8000 units of unfractionated heparin were used throughout the procedure. The dosing of unfractionated heparin was guided by the patient’s body weight as well as the activated clotting time.

The patient was asymptomatic throughout the procedure but started to have bilateral blurring of vision 30 minutes post-procedure, which worsened drastically over a period of 20 minutes to only being able to perceive light bilaterally. Apart from concomitant mild bitemporal headache, the patient had no other complaints. On examination, the patient scored 15 points on the Glasgow Coma Scale (GCS), blood pressure was 140/78 mmHg, heart rate was 86 beats per minute and was in sinus rhythm, respiratory rate was 20 breaths per minute, and oxygen saturation was 99% under room air. Pupils were 2 mm and reactive to light bilaterally, and there was no relative afferent pupillary defect. There were no other neurological deficits. Bedside echocardiography revealed preserved left ventricular ejection fraction without gross valvular abnormalities or left ventricular thrombus. Urgent ophthalmology review revealed normal anterior chambers and normal cup-to-disc ratio bilaterally on binocular indirect ophthalmoscopy. There were mild non-proliferative diabetic retinopathy changes in the left eye without other abnormalities otherwise. There were no cherry-red spots bilaterally to suggest central retinal artery occlusion. Nonetheless, the ophthalmology team decided to treat the patient as having bilateral central retinal artery occlusion, and intravenous acetazolamide was administered.

**Figure 1:**
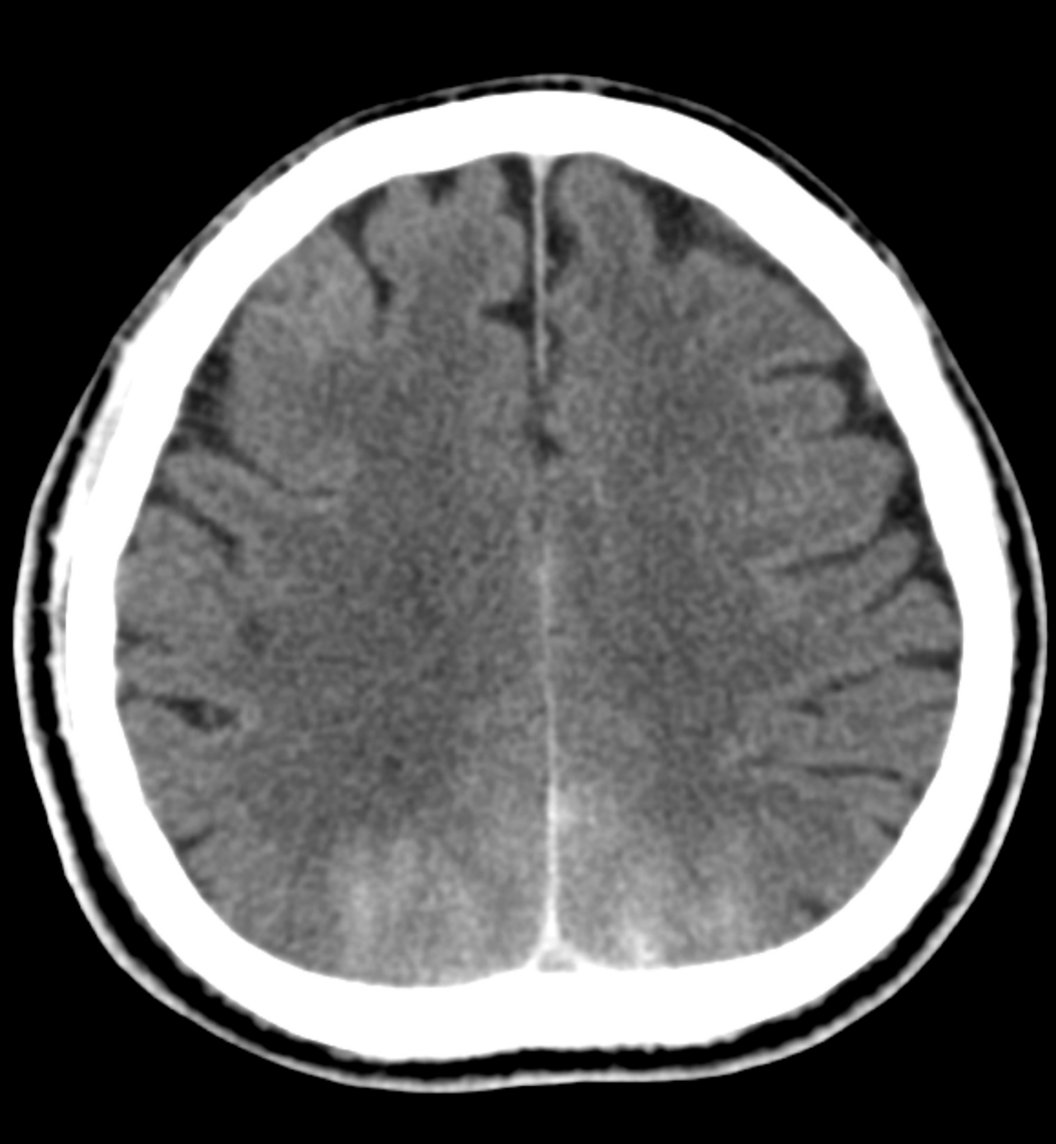
An urgent non-contrasted computed tomography scan of the brain revealed hyperdensities in both occipital lobes, which were reported as acute subarachnoid bleed.

At this point, a differential diagnosis of transient cortical blindness secondary to contrast agent was also considered, and the patient was started on intravenous hydration with normal saline (0.9% sodium chloride) to assist in diuresis of contrast agent. A repeat renal function test revealed normal creatinine level just as it was prior to the procedure. An urgent non-contrasted CT scan of the brain revealed hyperdensities in both occipital lobes, which were reported as acute subarachnoid bleed (Figure 1).

**Figure 2:**
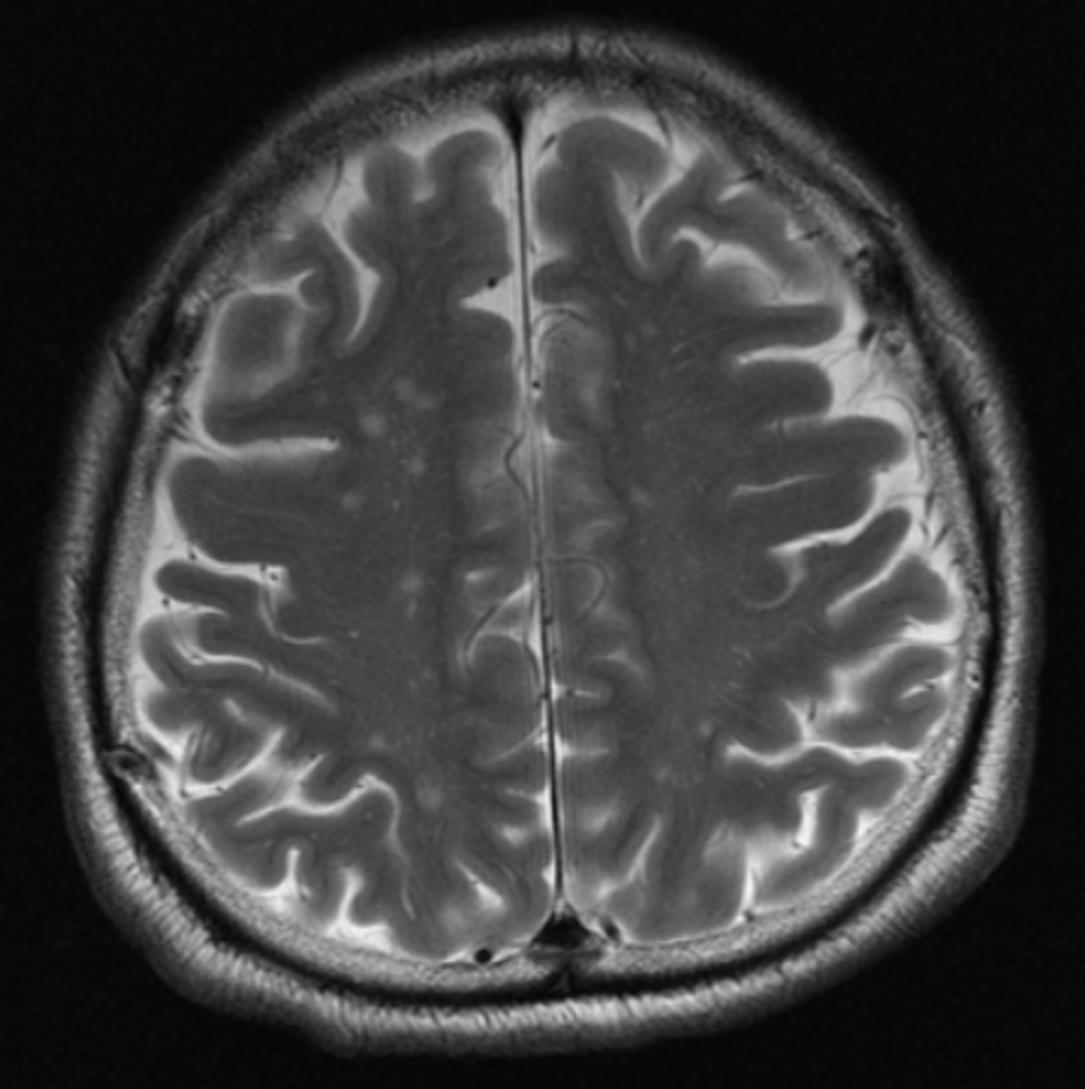
A magnetic resonance imaging scan of the brain, which was done at 17 hours after the index event of bilateral blurring of vision, revealed no evidence of intracranial bleed.

CT angiography of the brain revealed no evidence of aneurysm of intracranial arteries or arteriovenous malformation, and an additional 100 mL of non-ionic intravenous contrast (iopromide) was administered during the procedure. Neurosurgical opinion was then sought in view of the finding of subarachnoid bleed. In view that the patient scored 15 points on the GCS and he did not have other neurological deficits apart from vision abnormalities, the decision by the neurosurgery team was to treat the patient conservatively with regular GCS monitoring and with a plan to repeat a non-contrasted CT scan of the brain if the patient’s GCS reduces with a view for possible surgical intervention if required. Dual antiplatelet therapy in the form of aspirin and clopidogrel was withheld. Eight hours after the onset of bilateral blurring of vision, the patient gradually started to regain vision bilaterally. A magnetic resonance imaging (MRI) scan of the brain, which was done at 17 hours after the index event of bilateral blurring of vision, revealed chronic lacunar infarcts in both centrum semiovale, both corona radiata, left thalamus, and left putamen with no evidence of intracranial bleed (Figure 2) or acute infarct.

**Figure 3:**
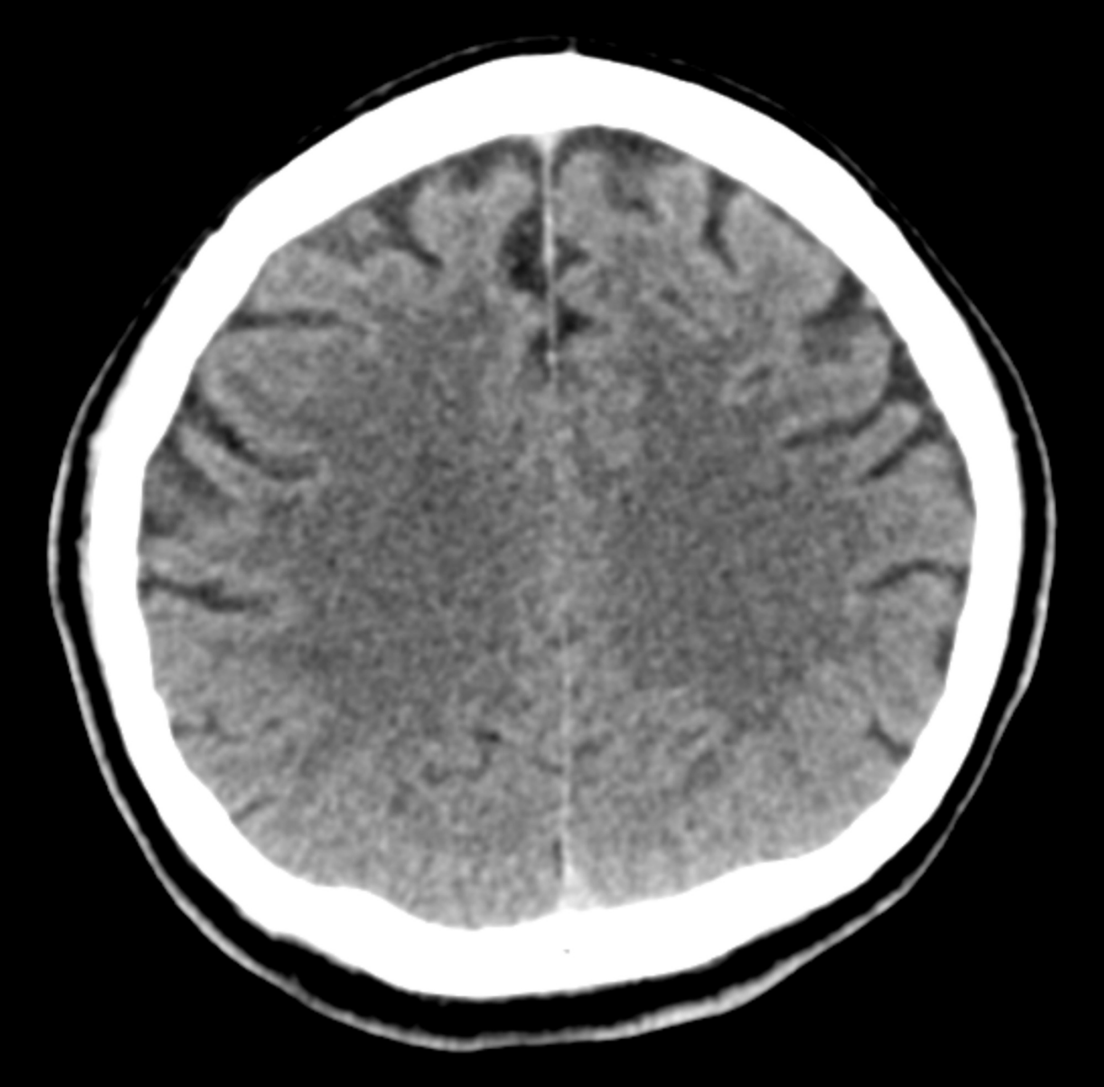
A repeat non-contrasted computed tomography scan of the brain 15 days after the index event revealed complete resolution of contrast material from the occipital lobes.

The neurology team was consulted, and given the absence of intracranial bleeding and acute stroke on the MRI scan of the brain, the consensus was to recommence dual antiplatelet therapy. By 20 hours after the index event, the patient had completely regained his vision bilaterally. A repeat ophthalmology review revealed objective improvement in the patient’s vision bilaterally, and he was discharged well two days later. A repeat non-contrasted CT scan of the brain 15 days after the index event revealed complete resolution of contrast material from the occipital lobes (Figure 3). When being reviewed at one month, three months, and six months after discharge, the patient reported that he had no vision abnormalities and was completely angina-free. 

## Discussion

Transient cortical blindness is a rare complication of coronary angiography. Transient cortical blindness is better recognized as a complication of cerebral and vertebral angiography with an incidence of 0.3% to 1%, but the rate can be as high as 4% when hyperosmolar iodinated contrast agents are used [2]. However, both ionic and non-ionic contrast agents are known to cause transient cortical blindness and do not seem to be volume-dependent [3]. The usage of modern, non-ionic, and low osmolarity iodinated contrast does not entirely prevent transient cortical blindness from happening, as has been illustrated by Yazici et al., who reported a case of transient cortical blindness after cardiac catheterization with iobitridol [4]. Transient cortical blindness is also listed as a possible complication in the product specification of non-ionic, low osmolarity iodinated contrast agents. The actual mechanism that causes transient cortical blindness after usage of contrast agents remains speculative to this very day despite it being first reported in the literature in the year 1970 [5]. One possible mechanism responsible for causing transient cortical blindness is a toxic reaction as the contrast agent penetrates the brain parenchyma, caused by an acute disruption of the blood-brain barrier [6]. Intraarterial contrast material apparently penetrates the blood-brain barrier by opening tight capillary junctions or enhancing endothelial pinocytosis. It then enters the cerebral cortex and adversely affects neuronal membranes [7].

Transient cortical blindness happens more frequently in the occipital cortex as opposed to other areas of the cortex, and the reason for this is possibly due to the sympathetic innervation of the occipital cortex being not as extensive and complete as in the carotid arterial system [3]. Most of the patients in whom transient cortical blindness has been reported have undergone angiography of bypass grafts. It is likely that, in these patients, direct injection of contrast into the vertebral artery occurs during angiography of the internal mammary artery conduit. The patients' prolonged supine posture might also play a role in the intracranial enhancement of the contrast solution in the occipital region [4]. As for our case, the total duration of the procedure was 160 minutes.

Transient cortical blindness after an invasive cardiac intervention confers a significant degree of anxiety and stress to both the patient and the treating doctors. The patient will undergo an anxious waiting period to see if his or her vision recovers and the treating doctors will be in a dilemma regarding the pathway of management, especially in the initial period after the index event of blurring of vision as at that juncture, transient cortical blindness secondary to contrast agent is, at best, a differential diagnosis. Other pertinent differential diagnoses, such as a central retinal artery occlusion or cerebral infarction secondary to embolization during the procedure, remain high on the list of suspicion. Very often, patients with sudden onset of bilateral blurring of vision after an invasive cardiac intervention will be managed by various subspecialty teams. It is not uncommon to have different ideas, opinions, and management methods among the different subspecialty teams. Therefore, it is imperative that all different subspecialty teams act promptly, in tandem, and be in constant communication to arrive at a conclusive, definitive, and unified clinical decision that is in the best interest of the patients.

The finding of the initial non-contrasted CT scan of the brain was an acute subarachnoid bleed in both occipital lobes; however, an MRI scan of the brain, which was done later, revealed no evidence of intracranial bleed. Subarachnoid bleed and leaked contrast can be differentiated by analyzing the anatomical location of the hyperdensity and the signal attenuation. Blood is located in the subarachnoid space with an attenuation of 40 to 60 Hounsfield units, while leaked contrast is located within the cortex and has an attenuation of 80 to 160 Hounsfield units. An MRI scan can be used for this purpose of differentiation [3]. Thus, we recommend using the MRI scan as the initial imaging modality of choice when one suspects the presence of transient cortical blindness due to a leaked contrast agent, given its superiority over a non-contrasted CT scan. Performing an MRI scan early after coronary stent implantation has been proven to be safe [8].

Based on the initial findings of the non-contrasted CT scan of the brain, dual antiplatelet therapy was withheld. That action was potentially detrimental as our patient had just undergone stenting of the left circumflex artery with a drug-eluting stent and prolonged withholdment of antiplatelet therapy drastically increases the risk of stent thrombosis, especially in the initial period after implantation of a drug-eluting stent. In our case, dual antiplatelet therapy in the form of aspirin and clopidogrel was recommenced as soon as the MRI scan of the brain ruled out an intracranial bleed. Therefore, various subspecialty teams that are likely to be involved in the care of patients with transient cortical blindness secondary to contrast agent will need to be aware of the existence of this rare and devastating albeit benign and self-limiting condition to avoid delay in instituting the accurate treatment plan and to avoid subjecting the patients to unnecessary medical or surgical procedures.

## Conclusions

We have highlighted a case of transient cortical blindness after coronary angiography, bypass graft angiography, and coronary angioplasty, which was secondary to the contrast agent. This condition, albeit rare, causes a significant amount of distress to both the patient and the treating doctors. It is hoped that this condition is better recognized by a variety of different subspecialty teams to ensure that prompt and appropriate investigation and management plans can be formulated and provided.
